# *Forkhead box C2* Promoter Variant c.-512C>T Is Associated with Increased Susceptibility to Chronic Venous Diseases

**DOI:** 10.1371/journal.pone.0090682

**Published:** 2014-03-07

**Authors:** Sumi Surendran, Athira Girijamma, Radhakrishnan Nair, Kalpana S. Ramegowda, Divya H. Nair, Jissa V. Thulaseedharan, Ravikumar B. Lakkappa, Giridhar Kamalapurkar, Chandrasekharan C. Kartha

**Affiliations:** 1 Rajiv Gandhi Centre for Biotechnology, Thiruvananthapuram, Kerala, India; 2 St. Thomas Institute of Research on Venous Diseases, Changanassery, Kerala, India; 3 Sri Jayadeva Institute for Cardiovascular Sciences & Research, Bangalore, India; 4 Achutha Menon Centre for Health Science Studies, Sree Chitra Tirunal Institute for Medical Sciences and Technology, Thiruvananthapuram, Kerala, India; 5 Kempegowda Institute of Medical Sciences, Bangalore, India; University Hospital Medical Centre, Germany

## Abstract

Chronic venous disease (CVD) is one of the most prevalent yet underrated disorders worldwide. High heritability estimates of CVD indicate prominent genetic components in its etiology and pathology. Mutations in human forkhead box C2 (*FoxC2*) gene are strongly associated with valve failure in saphenous and deep veins of lower extremities. We explored the association of genetic variants of *FoxC2* as well as *FoxC2* mRNA and protein expression levels with CVD of lower limbs. We systematically sequenced the single coding exon, 5′ and 3′ flanking regions of *FoxC2* gene in 754 study subjects which includes 382 patients with CVD and 372 healthy subjects. Four novel and three reported polymorphisms were identified in our cohort. Three variants in 5′ flanking region and one in 3′ flanking region of *FoxC2* gene were significantly associated with CVD risk. *FoxC2* mRNA in vein tissues from 22 patients was 4±1.42 fold increased compared to saphenous veins from 20 normal subjects (p<0.01). FoxC2 protein was also significantly upregulated in varicose veins compared to control samples. The c.-512C>T (*rs34221221: C>T*) variant which is located in the *FoxC2* putative promoter region was further analyzed. Functional analysis of c.-512C>T revealed increased mRNA and protein expression in patients with homozygous TT genotype compared to heterozygous CT and wild CC genotypes. Luciferase assay indicated higher transcriptional activity of mutant compared to wild genotype of this variant. These findings suggested that c.-512C>T variant of *FoxC2* was strongly associated with susceptibility to CVD and also that this variant resulted in FoxC2 overexpression. To obtain a mechanistic insight into the role of upregulated FoxC2 in varicosities, we overexpressed *FoxC2* in venous endothelial cells and observed elevated expression of arterial markers *Dll4* and *Hey2* and downregulation of venous marker *COUP-TFII*. Our study indicates altered FoxC2-Notch signaling in saphenous vein wall remodeling in patients with varicose veins.

## Introduction

Chronic venous disease (CVD) of the lower extremities is one of the most prevalent diseases worldwide though the prevalence estimates differ extensively due to the different disease evaluation methods. CVD comprises of visible venous disorders which are not associated with an identifiable mechanism of venous dysfunction [1]. They are manifested by a variety of signs ranging from telangiectasis and varicose veins to venous ulceration. CVD is generally termed as varicose veins, that being the most common form of clinical manifestation. The great saphenous vein and its tributaries are the anatomic sites frequently affected in CVD of lower limbs [2]. Structural failures of vein such as valve weakness or vein wall dilatation may result in venous retrograde flow in limb leading to distal high venous pressure causing CVD. The primary events resulting in valvular incompetence and primary vein wall changes are not yet elucidated.

Several risk factors contribute to the progression of CVD [3], [4], [5]. The major risk factors reported are age, sex, pregnancy, family history and life style factors such as occupations which demand prolonged-standing. Evaluations of family history of CVD revealed a high and consistent heritability estimate in this disease [6], [7]. Reports suggest that a risk of developing CVD for children with unaffected parents was only 20%. The risk with one affected parent is 25–62% and with both parents suffering with CVD the risk is 90% [6]. These data suggest the presence of genetic components in developing CVD, yet the precise genetic nature and genes involved in the pathogenesis of CVD is not known.

A twin cohort study indicated a link between varicose veins to microsatellite marker D16S520 on chromosome 16 [8]. This chromosomal region contains several genes coding for forkhead box family of proteins such as FoxC2 and FoxF1. *FoxC2* gene (FKHL14, MFH1) is located 80 kb distant from this marker. It was also reported that homozygous null mice of FoxC2 (*foxc2* ^−/−^) had abnormal lymphatic vascular patterning and malfunctioned blood vessels [9]. Even if it is well proved that FoxC2 is a transcription factor involved in cardiovascular development signaling [10] and lymphangiogenesis [11], its exact mode of action in vascular development is yet to be elucidated.

*FoxC2* gene variants are strongly associated with lymphedema distichiasis (LD) syndrome where majority of patients develop varicose veins [12]. *FoxC2* gene is also implicated in the pathogenesis of saphenous vein and deep vein reflux [13]. Yet there have been no further studies on *FoxC2* genetic variants in patients with varicose veins. We investigated the role of *FoxC2* genetic variants in the development of CVD of lower limbs in a case-control study. We quantified mRNA and protein expression level of *FoxC2* gene in saphenous vein from patients with varicose veins and healthy subjects. FoxC2 expression was highly upregulated in varicose vein tissues compared to normal control veins. Our results demonstrate significant correlation between c.-512C>T, a promoter variant of *FoxC2* and the expression levels of *FoxC2* mRNA and protein in CVD of lower limbs. FoxC2 overexpression in vein endothelial cells *in vitro* led to the upregulation of arterial markers such as *Hey2* and *Dll4* and the downregulation of venous marker, COUP TFII.

## Materials and Methods

### Ethics statement

The study was approved by the human ethics committees of Rajiv Gandhi Centre for Biotechnology, Thiruvananthapuram and all collaborating hospitals. Samples were collected from patients and healthy subjects after obtaining informed written consent.

### Subjects and Specimen Collection

382 patients with CVD and 372 control subjects were recruited for this study. Whole blood samples (5 ml) were collected from 360 patients (age - 18 to 60 years) with CVD from St.Thomas Hospital, Kerala, India. Diagnosis of CVD was based on physical examination and Doppler ultrasound test. CVD resulting from obstructions such as neoplasm were excluded from the study. Differential diagnosis was performed by an experienced vascular surgeon and presence of distichiasis was ruled out by an ophthalmologist. Patients with type 2 diabetes mellitus were also excluded since genetic variants of *FoxC2* have been reported to result in susceptibility to diabetes mellitus. Blood samples were collected from age and gender matched 352 healthy controls with no known family history for CVD.

For tissue level expression analysis, varicose vein tissue samples were collected from 22 patients admitted for treatment of CVD by operative treatments at Kempegowda Institute of Medical Sciences, Bangalore, India. Saphenous control vein samples from 20 patients who underwent coronary artery bypass graft surgery at Sri Jayadeva Institute for Cardiovascular Sciences & Research, Bangalore, India were also collected for the study. Whole blood samples were also collected from these 22 patients and 20 controls for sequencing assays. Relevant data regarding the clinical characteristics of patients were collected from medical records of the hospitals participating in the study.

### Data analysis

Demographic data of all study participants and information regarding symptoms such as pain, itching and throbbing sensation in legs and clinical signs such as hemorrhage, lower limb oedema, hyperpigmentation, thrombophlebitis, cellulitis and ulceration were collected for each patient from medical records. Family history, occupational and lifestyle data were collected to examine their influence in aggravating disease manifestation.

Disease phenotypes were categorized according to CEAP (clinical severity, etiology, anatomy and pathophysiology) classification system [14]. Varicose veins without odema or pigmentation were classified under C2. Only 2.9% of all our patients were in CEAP Class 3 in which varicose vein with oedema alone are found. The patients in this study were mostly from CEAP Class 4, 5 and 6 who presented various clinical signs such as pigmentation, ulceration along with oedema due to CVD.

### Genomic DNA and mRNA extraction

Genomic DNA from whole blood samples was extracted using QIAamp DNA blood mini kit (Qiagen, USA) according to the manufacturer's instructions. Genomic DNA and mRNA from vein tissues were extracted by All Prep DNA/RNA/Protein mini kit (Qiagen, USA). Quantification and purity of DNA and mRNA was measured by nanodrop-1000 spectrophotometer (ThermoScientific, USA) at 260 nm. RNA was further treated with DNase1 (Ambion, USA) for removing any DNA contamination.

### PCR DNA sequencing

A touch-down PCR was performed to amplify the single coding exon, 3 kb of 5′ flanking and 200 bp of 3′flanking region which includes the 5′ and 3′ untranslated regions (UTR) of *FoxC2* gene from DNA of patients with CVD and healthy subjects. Nine primer pairs (Table S1) to amplify overlapping regions of *FoxC2* gene and flanking regions were designed using Primer Premier 5 software (PREMIER Biosoft International, USA). PCR conditions were as follows: Initial denaturation for 5 min at 96°C, 20 cycles of denaturing at 96°C for 30 sec, annealing at 70°C for 40 sec with a touchdown of 0.5°C per cycle and extension at 72°C for 1.5 min. This was followed by 20 cycles at same conditions except that annealing was at 60°C for 40 sec. PCR products were purified using gel band elution kit (GE healthcare, USA). DNA sequencing was carried out on an ABI 3100 DNA analyzer with Bigdye terminator chemistry (Applied Biosystems, USA).

### Gene expression analysis of *FoxC2* by qRT-PCR

Total RNA (500 ng) from each tissue sample was subjected to reverse transcription with oligodT, dNTPs, and M-MLV reverse transcriptase (Promega, USA). Primers for *FoxC2* and *GAPDH* genes were designed for real time PCR analysis (Table S2). Quantitative RT-PCR was carried out as reported earlier [15]. The temperature conditions were as follows: 48°C, 30 min; 95°C, 10 min; followed by 40 cycles of 95°C,15 s; and 60°C, 1 min and analyzed using ABI Prism 7900HT sequence detection system (Applied Biosystems, CA). Values were normalized with GAPDH mRNA levels. A single peak was observed in the dissociation curve for both genes confirming the specificity of PCR products. Real time mRNA fold change was calculated by the formula, 2^−ΔΔCt^ [2^(Ct value of target gene-Ct value of control gene)^].

### FoxC2 protein expression analysis by western blot

Frozen vein tissues were homogenized and incubated in ice-cold RIPA buffer with protease inhibitor cocktail for 90 minutes followed by centrifugation at 15,000 *g* for 20 min at 4°C to collect the supernatant. Proteins were estimated by using Bradford reagent (Bio Rad, USA). Protein extracts (30 µg) were subjected to 12% SDS–PAGE and electro transferred to a Hybond C Extra membrane (Amersham, USA) as per the wet transfer procedure (Bio-Rad Mini Protean II). Membranes were blocked for 1 hour at room temperature in PBS containing 0.5% Tween-20 and 5% BSA, and incubated overnight with anti- FoxC2 antibody (1 µg/ml) at 4°C (Abcam, UK). Membrane was washed with TBS–Tween-20 and incubated with the 1∶10000 dilution of secondary antibody to rabbit IgG - H&L (HRP) (Abcam, UK) for 1 h at room temperature. Protein band was developed by enhanced chemiluminescence (ECL Plus Western Blotting detection system, GE Healthcare). The membrane was re-probed with anti GAPDH antibody (Abcam, UK) for normalization of expression. The densities of immunoreactive bands were quantitated by the Quantity One 1-D image analysis software program (Bio-Rad, USA).

### Immunostaining of FoxC2 antigen in tissue specimens

5 µm paraffin embedded tissue sections were de-paraffinized in xylene and endogenous peroxidase activity was quenched with 3% H_2_O_2_ in methanol by incubating for 30 minutes. Sections were rehydrated through graded alcohols and antigen retrieval was performed by incubating in 10 mM sodium citrate at 90°C for 15 mins. Sections were washed with TBST and then blocked with 3% BSA for 20 mins. Slides were incubated with anti- FoxC2 antibody (Abcam cat # ab65141) diluted with TBS in 1∶100 ratio. Slides were washed thrice for 5 minutes in TBST and incubated for 1 hour with Horse raddish peroxidase conjugated anti rabbit antibody (Abcam cat # ab97051) diluted with TBS in 1∶200 ratio. After washing, slides were incubated with 3,3′-diaminobenzidine tetrahydrochloride (DAB) (Sigma) and immediately washed under tap water after the color development and were counter stained with haematoxylin. Slides were DPX mounted and observed under light microscope (Nikon, Japan).

### Cloning and reporter gene assay

To elucidate the potential effect of c.-512C>T variant of *FoxC2* gene in the promoter activity of gene, a luciferase reporter gene assay was performed. A 664 bp DNA sequence from the −614 bp to +50 bp from the transcription start site of *FoxC2* gene encompassing the putative promoter area was amplified from DNA with CC and TT genotype. Primers including KpnI and HindIII restriction sites (Table S2) were used for the amplification. Amplicons were cloned into the KpnI and HindIII sites of the pGL4-basic vector (Promega, USA) and the constructs were verified by DNA sequencing.

EA.hy926 [16] cells were used for expressing the cloned constructs. The cells were cultured in Dulbecco's Modified Eagle's Medium (DMEM) containing 10% FBS in a humidified CO_2_ chamber at 37°C. 0.8 µg of constructs such as pGL4-Basic, pGL4-*FoxC2* promoter (CC genotype) and mutant pGL4-*FoxC2* promoter (TT genotype) were transfected into cultured cell lines using Lipofectamine (Invitrogen, USA). Renilla luciferase construct (phRL-TK) was used as a control for transfection efficiency. After 48 h, each group of cells was lysed and luciferase assay was carried out using Dual Luciferase assay kit (Promega, USA) according to manufacturer's instructions and readings were recorded. Technical replicates were performed in triplicate and biological experiments were performed twice.

### *FoxC2* construct and transfection of EA.hy926 cells

*FoxC2* – pCAGIG construct was made by inserting *FoxC2* coding sequence into EcoRI and XhoI restriction sites of pCAGIG mammalian expression vector (Addgene plasmid 11159) [17]. EA.hy926 cells were plated into 6-well plates and the cells were allowed to adhere for 24 hours. Transfection of *FoxC2* -pCAGIG and control empty vector was performed using lipofectamine-2000 (Invitrogen, CA) according to the manufacturer's recommendation. The concentrations of constructs used were 1 µg per well. After 6 hours of transfection, 20% FBS supplemented DMEM medium was added. The assays were carried out 8 days post-transfection. Transfection was performed in triplicates and repeated twice. GFP positive cells were sorted on FACS Aria (Becton Dickinson) and used for further analysis.

### Total RNA isolation and qRT PCR analysis

Total RNA from untransfected and transfected EA.hy926 cells was isolated by Allprep RNA/protein kit (Qiagen, USA). After the reverse transcription reaction as described earlier, cDNA was used for quantitative real time PCR for *FoxC2*, *GAPDH*, *Hey2*, *Dll4, COUP TFII* and *Ephrin B4* gene expression. Primers sequences are given in table S2. The conditions for amplifying *FoxC2* and *GAPDH* are as described earlier. For assessing *Hey2, Dll4, COUP TFII* and *Ephrin B4* gene expression, the reactions were performed in triplicate in 96-well plates at 48°C, 30 min; 95°C, 10 min; followed by 40 cycles of 95°C, 15 s; and 61°C, 1 min. The real-time PCR products were re-confirmed by electrophoresis on 2% agarose gels. The amount of the target relative to GAPDH mRNA was expressed as 2 ^−(ΔCt)^.

### Statistical analysis

Hardy-Weinberg equilibrium was tested for a goodness-of-fit using a Chi square test. Chi-square test was used to investigate the possible association between polymorphisms and CVD in case-control studies. Student's t test was used to analyze the difference in luciferase, mRNA transcripts and protein expression levels. Information collected from answered questionnaires and medical records were entered into MS Excel and analyzed using SPSS 16 (SPSS Inc., USA). Differences between groups were considered significant for p values less than 0.05.

## Results

Patients with CVD (n = 382) and healthy subjects (n = 372) had a mean age of 48.17 (SD = 13.37) and 37.86 (SD = 13.63) respectively (p<0.001). 53.7% of patients were females and a positive association was seen between female gender and CVD (p = 0.035). A detailed analysis of patients and controls with respect to different age groups and gender is given in table 1. To reduce a possible interference of these confounding variables in the current polymorphism analysis, we used adjusted odds ratio (AOR) with 95% confidence intervals (CI) estimated by multiple logistic regression models in each analysis. Clinical features of the patients with CVD are given in table 2.

**Table 1:** Demographic distribution of patients with CVD and controls.

Variables	Controls (N = 372) n(%)*	Cases (N = 382) n(%)*	P-value	^a^OR	^b^95% CI
**Age group**					
< = 29 years	132 (35.5)	40 (10.5)		1	
30–39 years	102 (27.4)	69 (18.1)		2.23	(1.4,3.56)
40–49 years	60 (16.1)	93 (24.3)	<0.001	5.12	(3.17,8.27)
50–59 years	39 (10.5)	103 (27)		8.72	(5.23,14.53)
> = 60 years	39 (10.5)	77 (20.2)		6.52	(3.86,11)
**Sex**					
Males	201 (54)	177 (46.3)	0.035	1	
Females	171 (46)	205 (53.7)		1.36	(1.02,1.81)

* Percentages were taken from the column totals. Chi-square test for measure of association was used to derive p value. ^a^Odds ratio and ^b^95% confidence intervals of individual groups.

**Table 2:** Clinical features in patients with CVD.

Variables	N = 382 n (%)*
Family history	257 (67.30)
Bleeding	29 (7.60)
Thrombophlebitis	3 (0.80)
Cellulitis	5 (1.30)
LL oedema	89 (23.30)
Pigmentation	185 (48.40)
Ulceration	56 (14.70)
CEAP Class	
2	48 (12.60)
3	11 (2.90)
4	223 (58.40)
5	73 (19.10)
6	27 (7.10)

*Percentages were taken from the column totals.

### *FoxC2* genotypes and risk for developing chronic venous disease

Distribution of genetic variants in 5′, 3′ flanking regions and coding sequence of *FoxC2* gene in patients with CVD and healthy controls are presented in tables 3 and 4. Hardy Weinberg equilibrium was satisfied in the observed genotype frequencies for control group (p>0.05). Four novel (c.-2257G>A, c.-2647A>T, c.-2649_-2647dupTC and c.*126G>A) and three previously reported polymorphisms [c.-350G>T (*rs78816814*, NG_012025.1∶g.4736G>T), c.-512C>T (*rs34221221*, NG_012025.1∶g.4574C>T) and c.-1538A>G *(rs4843162*, NG_012025.1∶g.3548A>G)] were observed. After adjusting for other confounding factors (age and gender), a significantly increased risk for CVD was found in patients carrying c.-512C>T, c.-1538A>G, c.-2647A>T and c.*126G>A variants. Allelic frequencies of these four polymorphisms also differed significantly between patients with CVD and controls (Table 5). Only these four polymorphisms were included in further analysis.

**Table 3:** Association between *FoxC2* genotypes (reported) and CVD risk.

Genotypes	Patients n(%)*	Controls n(%)*	^a^ OR (95%Cl)	P-value	^b^ AOR (95%Cl)
**c.-350G>T** ^c^(*rs78816814*)					
GG	342 (89.5)	325 (87.37)	1 (referent)		
GT	37 (9.7)	46 (12.37)	0.76 (0.48,1.21)		
TT	3 (0.8)	1 (0.27)	2.85 (0.3,27.55)		
GT/TT	40 (10.5)	47 (12.6)	0.81 (0.52,1.27)	0.353	0.72 (0.44,1.17)
**c.-512C>T** ^c^(*rs34221221*)					
CC	69 (18.1)	118 (31.7)	1 (referent)		
CT	209 (54.7)	170 (45.7)	2.1 (1.47,3.01)		2.37 (1.48,3.80)
TT	104 (27.2)	84 (22.6)	2.12 (1.40,3.20)		2.44 (1.42,4.17)
CT/TT	313 (81.9)	254 (68.3)	2.11 (1.50,2.96)	<0.001	2.08 (1.43,3.02)
**c.-1538A>G** ^c^(*rs4843162*)					
AA	240 (62.8)	280 (75.27)	1 (referent)		
AG	100 (26.2)	90 (24.19)	1.3 (0.93,1.81)		1.22 (0.80,1.87)
GG	42 (11)	2 (0.54)	24.5 (5.87,102.27)		25.58 (5.78,113.22)
AG/GG	142 (37.2)	92 (24.7)	1.8 (1.32,2.47)	<0.001	1.8 (1.28,2.53)

*Percentages were taken from the column totals. Chi-square test for measure of association was used to derive p value. ^a^Odds ratio and 95% confidence intervals of individual polymorphisms. ^b^ Adjusted odds ratio and 95% confidence intervals is obtained adjusting for age group and sex in multiple logistic regression model. ^c^Polymorphism previously reported in the Entrez single nucleotide polymorphism database (dbSNP).

**Table 4:** Association between *FoxC2* genotypes (novel) and CVD risk.

Genotypes	Patients n(%)*	Controls n(%)*	^a^ OR (95%Cl)	P-value	^b^ AOR (95%Cl)
**c.-2257G>A**					
GG	162 (42.4)	135 (36.3)	1 (referent)		
GA	160 (41.9)	167 (44. 9)	0.8 (0.58,1.09)		
AA	60 (15.7)	70 (18.8)	0.71 (0.47,1.08)		
GA/AA	220 (57.6)	237 (63.7)	0.77 (0.58,1.04)	0.086	0.86 (0.63,1.19)
**c.-2647A>T**					
AA	129 (33.8)	294 (79.0)	1 (referent)		
AT	228 (59.7)	77 (20.7)	6.75 (4.85,9.40)		6.35 (4.33, 9.31)
TT	25 (6.5)	1 (0.3)	56.98 (7.64,425.01)		57.01 (7.07,459.79)
AT/TT	253 (66.2)	78 (21)	7.39 (5.33,10.26)	<0.001	6.69 (4.71,9.51)
**c.-2649_-2647dupTC**					
Dup negative	348 (91.1)	352 (94.6)	1 (referent)		
Dup TC	34 (8.9)	20 (5.4)	1.72 (0.97,3.05)	0.061	2.39 (1.25,4.59)
**c.** * **126G>A**					
GG	221 (57.9)	308 (82.8)	1 (referent)		
GA	161 (42.2)	64 (17.2)	3.51 (2.50,4.91)		4.31 (2.81,6.60)
GA/AA	161 (42.1)	64 (17.2)	3.51 (2.50,4.91)	<0.001	4.31 (2.81,6.60)

*Percentages were taken from the column totals. Chi-square test for measure of association was used to derive p value. ^a^Odds ratio and 95% confidence intervals of individual polymorphisms. ^b^Adjusted odds ratio and 95% confidence intervals is obtained adjusting for age group and sex in multiple logistic regression model.

**Table 5:** Association of allelic frequencies of *FoxC2* polymorphisms in patients with CVD and control subjects.

Variables	Controls n (%)*	Cases n (%)*	P value
**c.-512C>T**			
C	288(53.1)	278(47)	
T	254(46.9)	313(53)	0.04
**c.-1538A>G**			
A	370(80.1)	340(70.5)	
G	92 (19.9)	142(29.5)	0.001
**c.-2647A>T**			
A	371(82.6)	357(58.5)	
T	78(17.4)	253(41.5)	<0.001
**c.** * **126G>A**			
G	372(85.3)	382(70.3)	
A	64(14.7)	161(29.7)	<0.001

*Percentages were taken from the column totals. Chi-squared test for measure of association was used to derive p value.

To understand the collective effect of these four significant polymorphisms in the disease, we further classified study subjects into two groups. Subjects with none or either one *FoxC2* variant were combined in one group. The second group comprised of subjects with two or more polymorphisms in their *FoxC2* gene and flanking sequences. Notably, the second group had 7.20 fold (95% CI: 4.99, 10.39) risk for CVD compared to first group (table S3).

DNA was isolated from vein specimens and sequenced to check any genotypes discrepancy between whole blood samples and tissues of same patients. The genotype profiles obtained were similar in both the DNA samples from same patients.

### Correlation of *FoxC2* genotypes with *FoxC2* mRNA transcript levels

**Figure 1:**
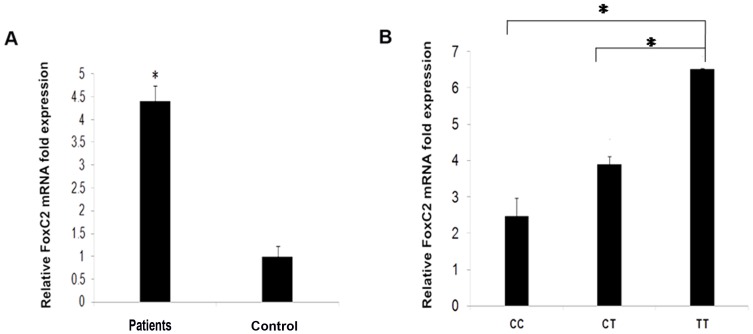
*FoxC2* mRNA expression in saphenous vein tissues from patients with CVD and healthy subjects. **(A)** Relative *Foxc2* mRNA fold increase in CVD tissues (n = 22) compared to control specimens (n = 20). **(B)** Relative *FoxC2* mRNA levels in vein tissues from patients carrying CC, CT and TT genotypes of c.-512C>T (rs34221221) variant. The difference in expression in patients with CT and TT genotypes were statistically significant compared to wild CC genotype (*p<0.05). Data shown are the mean ± SD.

*FoxC2* transcript expression was 4±1.4 folds increased in venous tissues from patients compared to normal subjects (p<0.05) (Figure 1a). Patients with homozygous mutant TT genotype had higher venous expression of *FoxC2* mRNA compared to patients carrying heterozygous CT genotype (p = 0.02) and wild CC genotype (p<0.001) (Figure 1b). The upregulation of *FoxC2* in tissue specimens (either at mRNA or protein level) was not significantly altered in patients who had all the four polymorphisms compared to four patients who carried TT genotype of c.-512C>T variant alone (p = 0.65) (Figure S2).

### Correlation of *FoxC2* genotypes with FoxC2 protein expression levels

**Figure 2:**
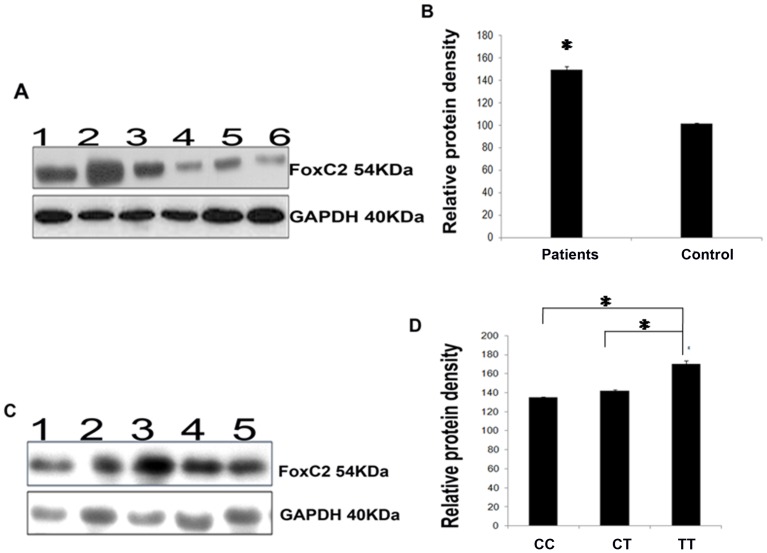
*FoxC2* protein expression in saphenous vein tissues from patients with CVD and healthy subjects. **(A)** Immunoblot analysis of FoxC2 protein in patients and controls, lanes 1–3 patient vein tissue protein homogenates, lanes 4–6 control saphenous vein protein homogenates. **(B)** Densitometric analysis of immunoreactive bands indicates an upregulation of FoxC2 protein in vein specimens from patients (n = 22) compared to controls (n = 20). **(C)** Immunoblot analysis of FoxC2 protein in patients carrying lane 1: CC, lane 2–3: TT and lane 4–5: CT genotypes. **(D)** Graph showing a correlation of FoxC2 protein expression associated with CC, CT and TT genotypes of c.-512C>T (rs34221221) genotype in patients with CVD. FoxC2 protein was significantly increased in venous tissues of patients carrying TT genotype than those with CC and CT genotypes of c.-512C>T variant (*p<0.01). Data shown are the mean ± SD.

Densitometry analysis of immunoblots indicated a significant upregulation of FoxC2 protein in varicosed tissues compared to control (Figures 2a and 2b). Correlation of densitometry results of *FoxC2* protein expression with *FoxC2* genotypes revealed significantly higher protein levels in patients carrying TT genotype compared to patients having heterozygous CT or wild CC genotypes (p<0.01) (Figures 2c and 2d).

### Immunostaining of FoxC2 antigen in tissue sections

**Figure 3:**
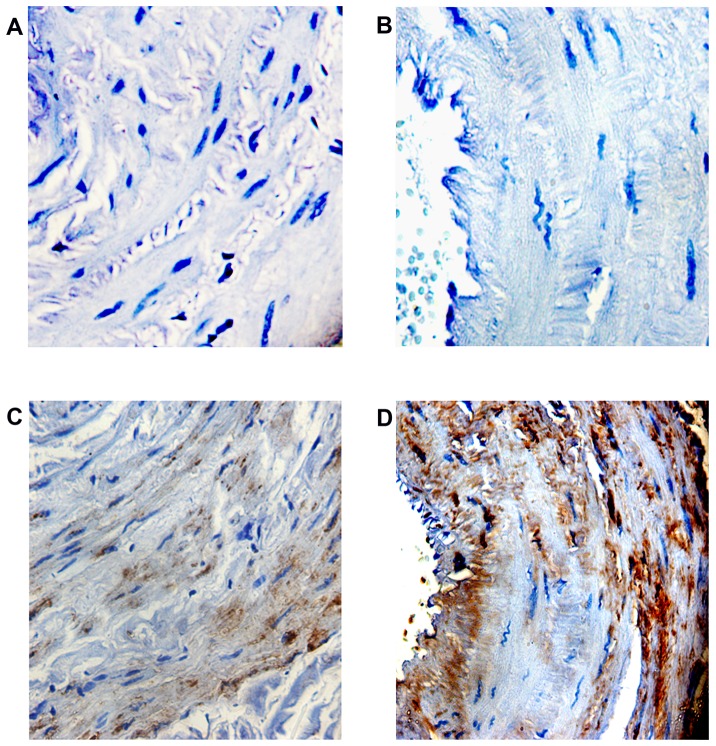
Photomicrograph demonstrating immunohistochemical staining with FoxC2 antibody in venous tissue sections from patients with varicose veins and controls. **(A)** Negative staining control in normal saphenous veins and **(B)** varicose vein tissues. **(C)** FoxC2 expression is localized to tunica media in normal venous tissue section. **(D)** FoxC2 is expressed all over intima and media of varicose vein tissues with high staining intensity, original magnification x 400.

To confirm the overexpression of FoxC2 in varicose veins we performed an immunostaining on tissue sections of varicose veins from 3 patients and control vein from 3 healthy subjects. There was a marked overexpression of FoxC2 protein in the tissue sections of varicose veins compared to control veins (Figure 3).

### Reporter gene assay

**Figure 4:**
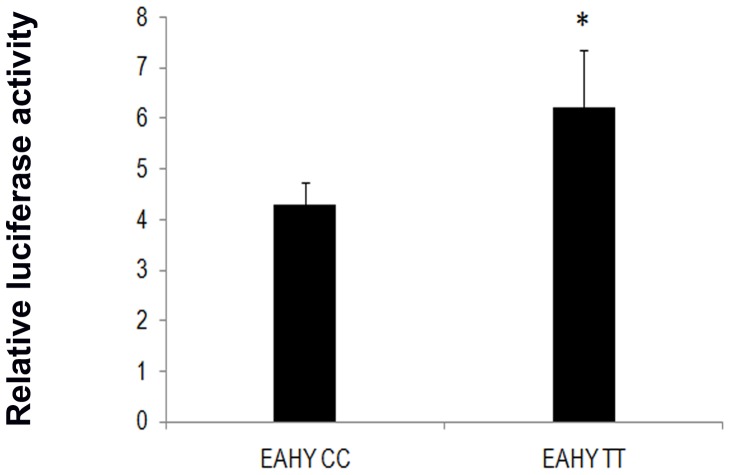
Effect of *FoxC2* variant c.-512C>T (rs34221221) on luciferase expression in EA.hy926 cells. Cell lines were transiently transfected with the FoxC2 promoter reporter plasmid containing CC genotype or TT genotype. The renilla luciferase plasmid was co-transfected with reporter constructs to normalize the luciferase activity. Transfection with mutant construct (pGL4-TT) demonstrated a slight increase in luciferase activity (*p = 0.051) compared to wild pGL4-CC construct. Data shown are the mean ± SD of technical triplicates of two biological experiments.

From our disease association results and transcript and protein expression analysis it was evident that c.-512C>T variant was related with altered mRNA and protein expression in vein tissues of CVD. We further examined if gene transcription was affected by this promoter polymorphism. As demonstrated in figure 4, EA.hy926 cells transfected with homozygous variant (pGL4-TT) constructs exhibited marginally increased luciferase activity (p = 0.051) compared to wild type constructs (pGL4-CC).

### Expression of *Hey2*, *Dll4*, *COUP TFII* and *Ephrin B4* in *FoxC2*-Ea.hy926 cell constructs

**Figure 5:**
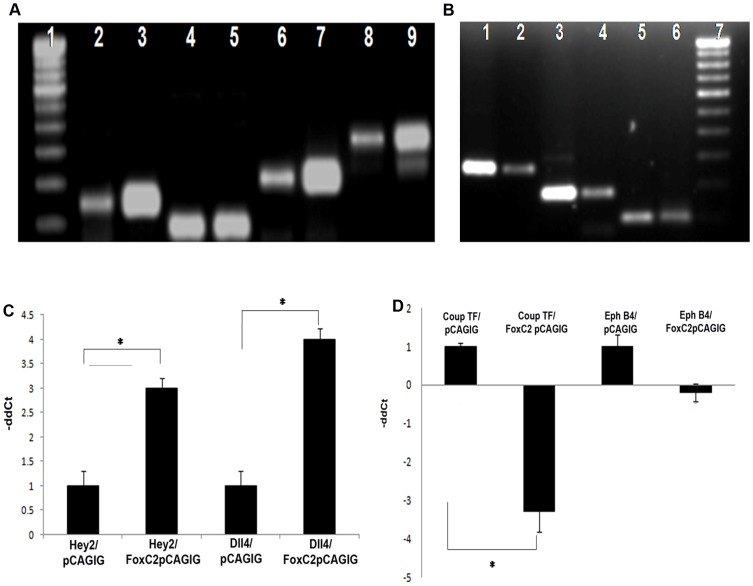
*FoxC2* based regulation of *Hey2*, *Dll4*, *COUP TFII* and *Ephrin B4* expression in EA.hy926 cells. **(A)** Gel image showing increased expression of *Dll4* and *Hey2* mRNA in EA.hy926 cells transfected with *FoxC2* –pCAGIG. Lane 1: 100 bp molecular ladder, Lane 2, 4, 6, 8: *FoxC2, GAPDH, Dll4* and *Hey2* mRNA levels in cells transfected with pGAGIG control vector. Lane 3, 5, 7, 9: *FoxC2, GAPDH, Dll4* and *Hey2* mRNA levels in *FoxC2*- pGAGIG construct transfected cells. **(B)** Gel image showing increased expression of *COUP TFII* and *Ephrin B4* mRNA in EA.hy926 cells transfected with *FoxC2* –pCAGIG. Lane 1, 3, 5: *Ephrin B4, COUP TFII* and *GAPDH* mRNA levels in cells transfected with empty pGAGIG control vector. Lane 2, 4, 6: *Ephrin B4, COUP TFII* and *GAPDH* mRNA levels in *FoxC2*- pGAGIG construct transfected cells, Lane 7: 100 bp molecular ladder **(C)** Relative mRNA levels of *Hey2* and *Dll4* measured by qRT-PCR. **(D)** Relative mRNA levels of *COUP TFII* and *Ephrin B4* measured by qRT-PCR. Values are mean±s.d. of triplicate experiments from 3 samples per each group. Statistical significance was determined by Student's t-test (*p<0.05 versus control).

Based on the reports of a potential FoxC2- Notch signaling [18], we focused on the expression patterns of Notch ligand *Dll4* and arterial marker *Hey2* in the *FoxC2* construct transfected Ea.hy926 cells. Expression of venous specific markers such as *COUP TFII* and *Ephrin B4* were also assessed. Ea.hy926 cells were transiently transfected with pCAGIG vector containing *FoxC2* construct (*FoxC2*-pCAGIG) or empty pCAGIG vector and the expression of *Hey2* and *Dlll4* genes were determined at mRNA levels. *FoxC2* activation upregulated (p<0.05) the expression of both these arterial fate specific markers in Ea.hy926 when compared to empty vector transfected cells (Figure 5). *FoxC2* overexpression resulted in a significant downregulation (p = 0.003) of *COUP TFII*, while *Ephrin B4* downregulation was not statistically significant.

## Discussion

Human *FoxC2* is a forkhead/winged helix transcription factor coding gene located on chromosome 16q24.1 (NCBI Reference Sequence No: NG_012025.1). FoxC2 is implicated in vascular development especially in arterial and lymphatic differentiation [19]. Foxc2 deficiency in mouse results in abnormal lymphatic patterning and failure of lymphatic valve formation [9]. Mutations in the *FoxC2* coding sequences were reported in patients with lymphoedema-distichiasis (LD) which is an autosomal dominant form of primary lymphoedema with majority of patients developing varicose veins in lower limbs [12]. Several studies have associated mutations in *FoxC2* gene with LD risk and suggested a role of *FoxC2* in the pathogenesis of varicose veins [12]. Mellor *et al* linked *FoxC2* mutations to venous valve failure and reflux using conventional colour Doppler duplex ultrasound in patients with lymphodema distichiasis [13]. The role of *FoxC2* gene however has not yet been well-defined in patients with varicose veins or CVD. We report for the first time a positive association between genetic variants of *FoxC2* and chronic venous diseases and a mechanistic insight on the role of FoxC2 in pathogenesis of CVD.

*FoxC2* polymorphisms and abnormal protein expression have been implicated with insulin sensitivity in patients with obesity and diabetes mellitus [20]. We hence excluded patients with diabetes and obesity from this study population to get an unbiased data of *FoxC2* polymorphism pattern in patients with CVD alone. Patients with lymphoedema distichiasis were also not included in our subjects.

We initially sequenced the 1.5 kb single coding exon of *FoxC2* gene from DNA isolated from whole blood samples of 382 patients with CVD and 372 control subjects. DNA sequencing revealed the presence of only two rare synonymous variants, c.354C>T (novel variation) and c.426G>A (*rs41312258*) with a frequency of 0.02 of 0.04 respectively. Hence these variations were not followed up for further studies. The analysis was extended to approximately 3 Kb 5′ untranslated flanking region of *FoxC2* gene as well as 200 bp of 3′ flanking region which also includes the 3′ UTR region of gene. Seven *FoxC2* polymorphisms were observed in patients with CVD and normal subjects. Two reported variants (c.-512C>T: *rs34221221* and c.-1538A>G: *rs4843162*) and two novel variants c.-2647A>T and c.*126G>A were found to be significantly associated with risk of disease.

Variants such as c.-2647A>T and c.-1538A>G were not further experimentally validated as they lacked the putative binding sites for transcription factors. Transcription factor binding affinity was evaluated by TF SEARCH version 1.3 computer program (http://www.cbrc.jp/research/db/TFSEARCH. html) [21]. C.*126G>A variant is located 126 bp downstream to translation termination codon and 35 bp downstream to 3′UTR sequence of *FoxC2* gene. C.*126G>A was consistently present in either heterozygous GA or wild GG genotypes but never in homozygous mutant AA genotype in our cohort. Prediction of microRNA/target duplexes for C.*126G>A variant was analyzed by miRNA prediction tools (http://bibiserv.techfak.uni-bielefeld.de/rnahybrid) [22] and miRBase database [23]. Even though a putative binding site for Has-mir-4732-5p was obtained at this variant's nucleotide position from miRBase database, further *in silico* analysis by RNAhybrid tool gave a very weak binding probability. C.-512C>T variant is present in the highly conserved proximal promoter of the *FoxC2* gene [20]. This variant can possibly alter transcription factor binding and subsequent gene expression and hence was selected for further tissue centric expression analysis.

*FoxC2* mRNA and protein were over expressed in vein tissues of patients with CVD compared to normal saphenous vein specimens. The *FoxC2* mRNA transcript and protein upregulation in vein tissues positively correlated with the presence of TT genotype of c.-512C>T polymorphism in all the patients with CVD. Our observations are in concordance with an earlier report that variations outside the forkhead domain of *FoxC2* result in a gain of function [24]. A slight increase in gene expression was observed with reporter luciferase assays using mutant construct (p = 0.051) which indicates the contribution of other polymorphisms and factors in this upregulation as well. Since this is an initial study with 754 subjects, further studies in multiple cohorts is essential to verify our conclusion.

*FoxC1 and FoxC2* transcription factors promote arterial specification during vascular development by acting upstream of Notch [19]. Arterial specific markers such as *Dll4* and *Hey2* were found overexpressed and venous marker *COUP TFII* was found downregulated in vein endothelial cells transfected with *FoxC2* overexpressing mammalian construct. Our observations support the earlier reports on Hey2 and Dll4 based inhibition of Coup TFII *in vitro* [25]. As Hey2 is an important regulator of smooth muscle proliferation [26], we assume an altered FoxC2- Notch signaling in vein wall thickening in varicose veins. While arterial markers, *Hey2* and *Dll4* expression was upregulated in RNA samples from patients with CVD and controls (Figure S1), venous markers did not show any differential expression in RNA samples from patients with CVD and controls (data not included).

Taken together, our results suggest c.-512C>T variant can contribute to the upregulation of FoxC2 in vein tissues. This possibly triggers an altered FoxC2- Notch signaling cascade which results in the remodeling of saphenous vein in patients with CVD.

## Supporting Information

Figure S1 ***Hey2*** **and** ***Dll4*** **mRNA expression in vein tissues of patients with CVD and healthy subjects.** Lane 1–3: *Hey2* in control saphenous vein, lane 4–6: *Hey2* in vein tissues from patients with CVD, lane 7: 100 bp molecular ladder, lane 8–10: *Dll4* in control saphenous vein, lane 11–13: *Dll4* in vein tissues from patients with CVD.
(TIF)

Figure S2 ***FoxC2*** **mRNA expression in vein tissues from patients with CVD.** Relative *FoxC2* mRNA levels in vein tissues from patients (n = 5) carrying all the four variants such as c.-512C>T, c.-1538A>G, c.-2647A>T and c.*126G>A and *FoxC2* mRNA levels in vein tissues from patients (n = 4) carrying only TT genotype of c.-512C>T (rs34221221) variant. The difference in mRNA expression in both groups was statistically insignificant (p = 0.65). Data shown are the mean ± SD in each group.
(TIF)

Table S1 **Primers used for PCR and sequencing of 5′ flanking region, 3′flanking regions and coding sequence of** ***FoxC2*** **gene.**
(DOC)

Table S2 **Primers used for quantitative real time PCR and luciferase reporter assay.**
(DOC)

Table S3 **Patients and controls categorized as first group with neither or any single polymorphism and second group with 2, 3 or more polymorphisms.** Percentages were taken from the column totals. Chi-square test for measure of association was used to derive p value. ^a^ Odds ratio and 95% confidence intervals of individual polymorphisms. ^b^ Adjusted odds ratio and 95% confidence intervals is obtained adjusting for age group and sex in multiple logistic regression model.
(DOC)
