# Supplementary figures and images for: Forkhead box C2 Promoter Variant c.-512C>T Is Associated with Increased Susceptibility to Chronic Venous Diseases
Source: PLoS One. 2014 Mar 7;9(3):e90682. doi: 10.1371/journal.pone.0090682 (PMC3946558; doi:10.1371/journal.pone.0090682)

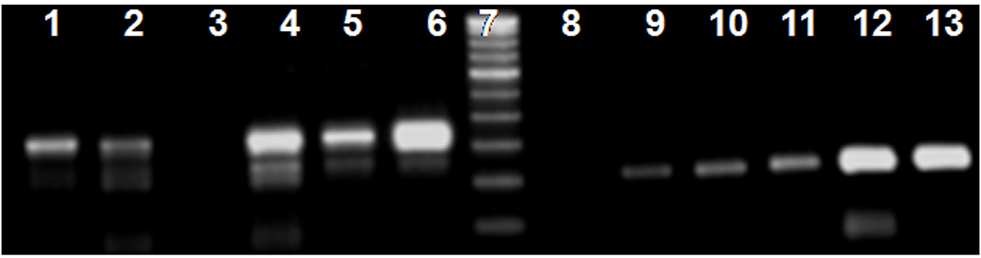

Supplement: Figure S1 — Hey2 and Dll4 mRNA expression in vein tissues of patients with CVD and healthy subjects. Lane 1–3: Hey2 in control saphenous vein, lane 4–6: Hey2 in vein tissues from patients with CVD, lane 7: 100 bp molecular ladder, lane 8–10: Dll4 in control saphenous vein, lane 11–13: Dll4 in vein tissues from patients with CVD. (TIF) [file pone.0090682.s001.tif]

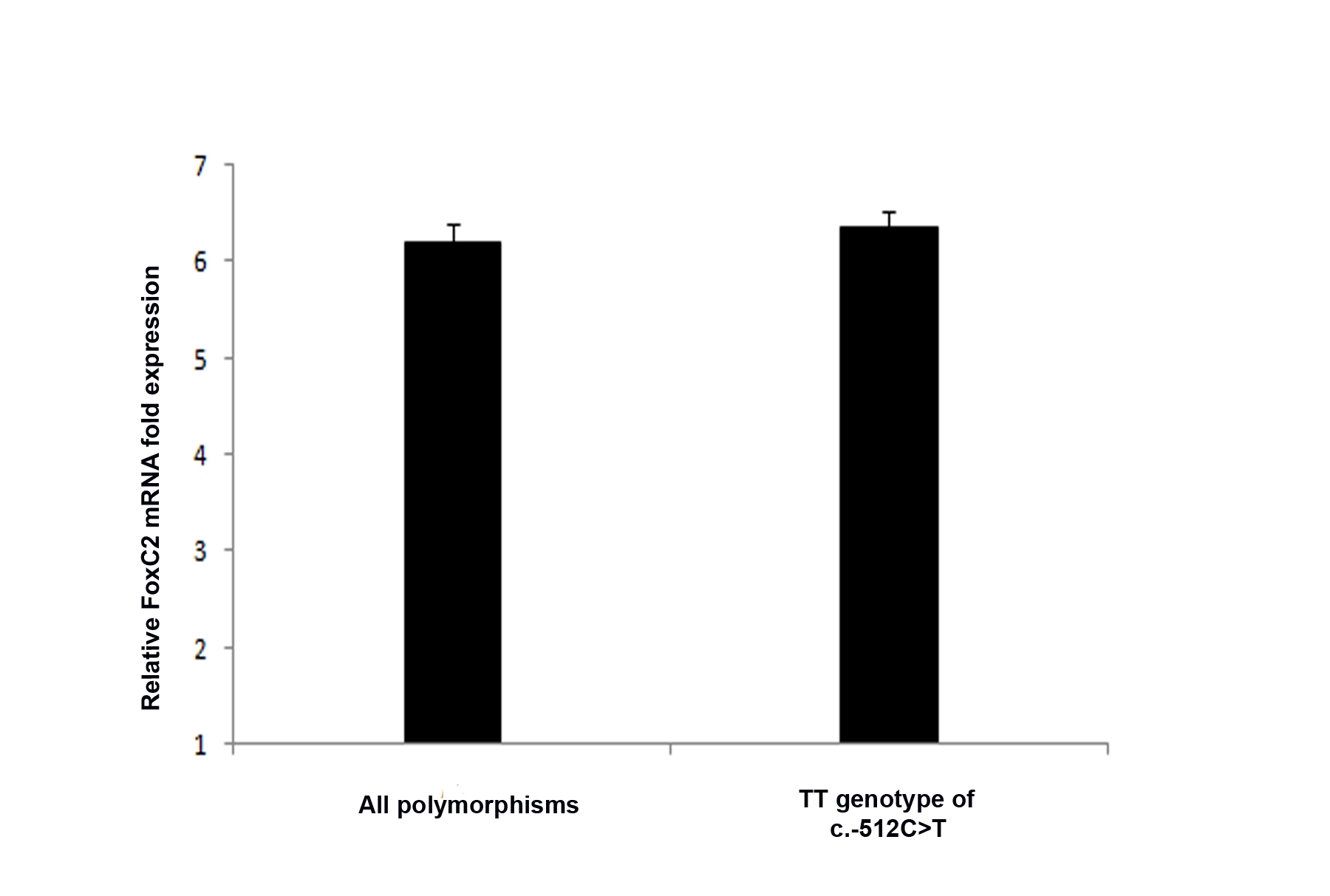

Supplement: Figure S2 — FoxC2 mRNA expression in vein tissues from patients with CVD. Relative FoxC2 mRNA levels in vein tissues from patients (n = 5) carrying all the four variants such as c.-512C>T, c.-1538A>G, c.-2647A>T and c.*126G>A and FoxC2 mRNA levels in vein tissues from patients (n = 4) carrying only TT genotype of c.-512C>T (rs34221221) variant. The difference in mRNA expression in both groups was statistically insignificant (p = 0.65). Data shown are the mean ± SD in each group. (TIF) [file pone.0090682.s002.tif]
