# Supplementary material for: Forkhead box C2 Promoter Variant c.-512C>T Is Associated with Increased Susceptibility to Chronic Venous Diseases
Source: PLoS One. 2014 Mar 7;9(3):e90682. doi: 10.1371/journal.pone.0090682 (PMC3946558; doi:10.1371/journal.pone.0090682)
Supplement: Table S1 — Primers used for PCR and sequencing of 5′ flanking region, 3′flanking regions and coding sequence of FoxC2 gene. (DOC) [file pone.0090682.s003.doc]

**Table S1: Primers used for PCR and sequencing of 5’ flanking region, 3’flanking regions and coding sequence of *FoxC2*** gene

| **Primer & 5’ position of each primer** | **Sequence (5’-3’)** |
| --- | --- |
| **F1; -2976**  **R1; -2384** | CCGGAGTCCCACTGAGCAAAC  CCCAGTTCTCAGCTCACAGGCAA |
| **F2; -2420**  **R2; -1685** | TGGGAGTTTAGGGTTGGGGCAGA  TGTTTTGTTGGGTGGGTAGGGTG |
| **F3; -1728**  **R3; -1061** | TCAACCGCTCACCCAGTCTTCC  TCCAGGCTCGGGGTCGTCAAG |
| **F4; -1147**  **R4; -533** | CTATGCACTCCGCTGCCTGGCT  AAAGTCTTCTTGCTGAAAGCGAGTT |
| **F5; -614**  **R5; -218** | CCCCGATTGGCGCCGACTC  TTTCAGCGGACCGGGCGGATC |
| **F6; -289**  **R6; +310** | CGCCGGGTCCTGGAGCCA  CGGTAGAAGGGGAAGCGGTC |
| **F7; +219**  **R7; +821** | GCCCTACAGCTACATCGCGCTCATC  CGACGTTCGCAGGGTCATGATGT |
| **F8; + 802**  **R8; +1313** | CCTGGCTTCAGCGTGGAGAA**C**AT**C**  GCTGCTGGGCCGCGAACGTGT |
| **F9; +1278**  **R9; +1683** | CAACCACAGCGGGGACCTGAACC  GCGGCGAATTAAAGTTACCTGC |
