# Supplementary material for: Forkhead box C2 Promoter Variant c.-512C>T Is Associated with Increased Susceptibility to Chronic Venous Diseases
Source: PLoS One. 2014 Mar 7;9(3):e90682. doi: 10.1371/journal.pone.0090682 (PMC3946558; doi:10.1371/journal.pone.0090682)
Supplement: Table S2 — Primers used for quantitative real time PCR and luciferase reporter assay. (DOC) [file pone.0090682.s004.doc]

**Table S**2: Primers used for quantitative real time PCR and luciferase reporter assay.

| **Primer** | **Sequence (5’-3’)** |
| --- | --- |
| **qRT FoxC2F**  **qRT FoxC2R** | TTCGCGGCCCAGCAGCAA  AGAGAGGCGGCGTGGATC |
| **GAPDHF**  **GAPDHR** | TGCACCACCAACTGCTTA  GGCATGGACTGTGGTCAT |
| **Fox KpnI**  **Fox HindIII** | CGGGGTACCCCGCCCCGATTGGCGCCGACTCCT  CCCAAGCTTGGGGGGCGTTGGGGTCGGACAC |
| **Hey2F**  **Hey2R** | GCAACAGGGGGTAAAGGCTAC  GAAGTTGTGGAGAGGCGAC |
| **Dll4F**  **Dll4R**  **COUP TFII F**  **COUP TFII R**  **Ephrin B4F**  **Ephrin B4R** | ATGTGTCATTGCCACGGAGGTAT  AGGTGTGGAAGGGTATTGCATCTC  GAGACGGGAAGCGGTGCAGAG  GCATGCATTGGCTGCCGAAG  CTCTGCTGAGGACCTGCTCCGAATC  ACTCCTCACCCCACGGGCTCAA |
