# Supplementary material for: Forkhead box C2 Promoter Variant c.-512C>T Is Associated with Increased Susceptibility to Chronic Venous Diseases
Source: PLoS One. 2014 Mar 7;9(3):e90682. doi: 10.1371/journal.pone.0090682 (PMC3946558; doi:10.1371/journal.pone.0090682)
Supplement: Table S3 — Patients and controls categorized as first group with neither or any single polymorphism and second group with 2, 3 or more polymorphisms. Percentages were taken from the column totals. Chi-square test for measure of association was used to derive p value. a Odds ratio and 95% confidence intervals of individual polymorphisms. b Adjusted odds ratio and 95% confidence intervals is obtained adjusting for age group and sex in multiple logistic regression model. (DOC) [file pone.0090682.s005.doc]

**Table S3: Patients and controls categorized as first group with neither or any single polymorphism and second group with 2, 3 or more polymorphisms.**

|  | **Controls (N=372)**  **(N, %)*** | **Cases (N=382)**  **(N, %)*** | **OR^a^**  **(95%Cl)** | **p-value** | **AOR^b^ (95%Cl)** |
| --- | --- | --- | --- | --- | --- |
| **0 or 1 polymorphism**  **2, 3 or all polymorphisms** | 219(58.90)  153(41.10) | 64(16.80)  318(83.20) | 1  7.11(5.07,9.98) | <0.001 | 1  7.20 (4.99,10.39) |

*Percentages were taken from the column totals. Chi-square test for measure of association was used to derive p value. ^a^ Odds ratio and 95% confidence intervals of individual polymorphisms. **^b^** Adjusted odds ratio and 95% confidence intervals is obtained adjusting for age group and sex in multiple logistic regression model.
